# Supplementary material for: Pre-40S ribosome biogenesis factor Tsr1 is an inactive structural mimic of translational GTPases
Source: Nat Commun. 2016 Jun 2;7:11789. doi: 10.1038/ncomms11789 (PMC4895721; doi:10.1038/ncomms11789)
Supplement: Supplementary Information — Supplementary Figures 1-4, Supplementary Methods and Supplementary References. [file ncomms11789-s1.pdf]

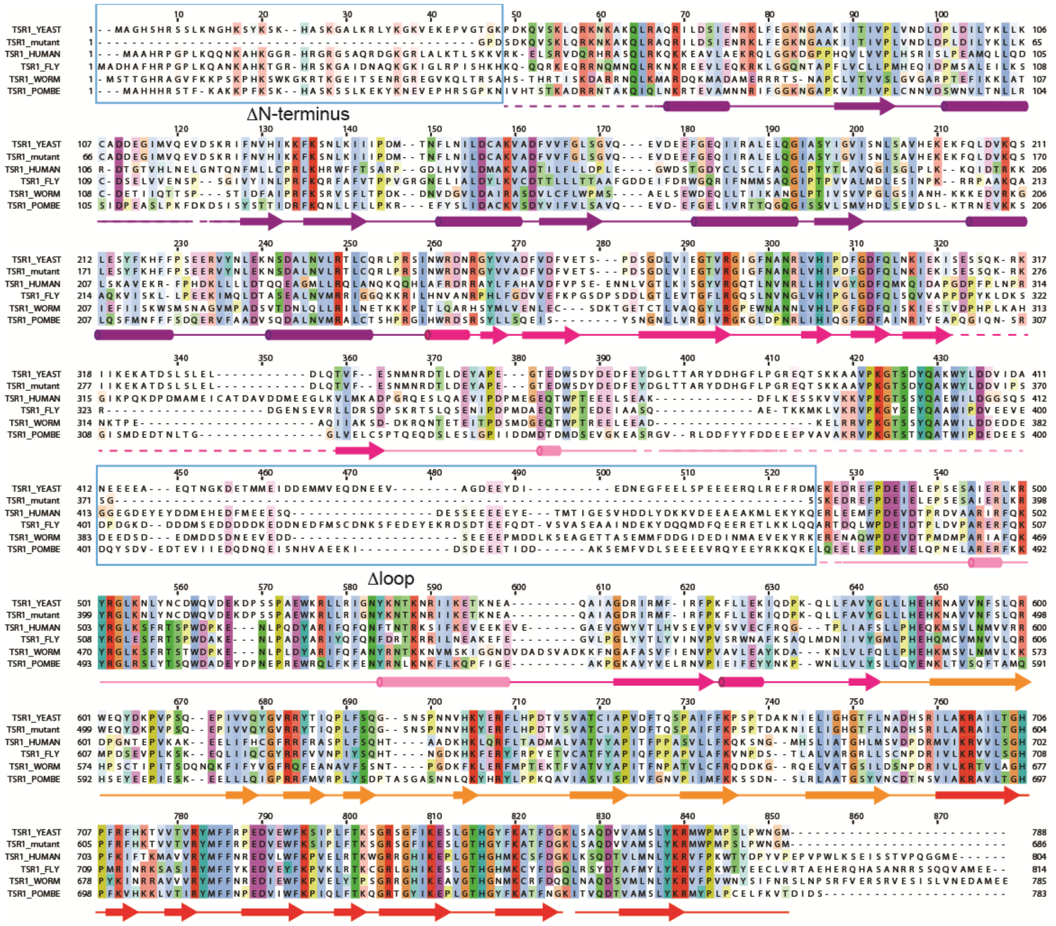

Supplementary Figure 1. Sequence conservation of Tsr1

A multiple sequence alignment of Tsr1 across metazoa and fungi showing the position of deleted sequences (blue boxes). Secondary structure elements are colored by associated domain, according to the schema in Fig. 1. Regions of the chain not visible in electron density maps are depicted with a dotted line.

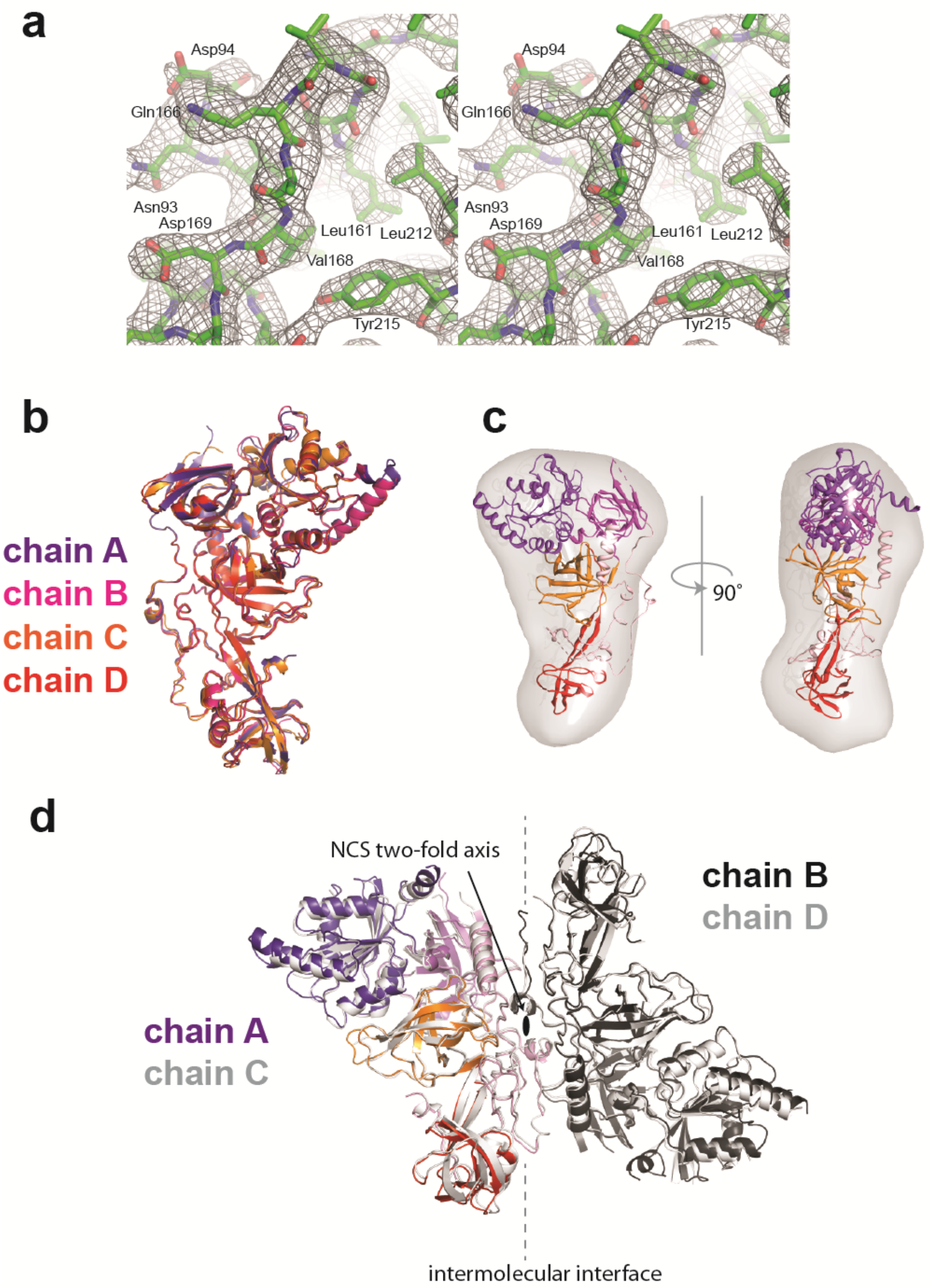

Supplementary Figure 2. Structural analysis of Tsr1

(**a**) Stereo view of the electron density map in domain I of chain A. (**b**) A global superposition of all four chains of Tsr1 found in the asymmetric unit of the crystals. All chains are depicted as cartoons and colored purple (A), pink (B), orange (C) and red (D). (**c**) Tsr1 coordinates fitted to the previously published negative stain EM map of Tsr1 (EMD1922), indicating that both the crystal and negative-stained forms of Tsr1 have the same shape. (**d**) In the asymmetric unit of Tsr1 crystals, chains A and B form an equivalent dimer to chains C and D. These pairs of chains are shown superposed with chain A colored according to the schema in figure 1, chain B in black and chains C and D in gray. The view is down the two-fold axis of the dimer and is related to the “back” view of chain A with a ~30º rotation around the x-axis.

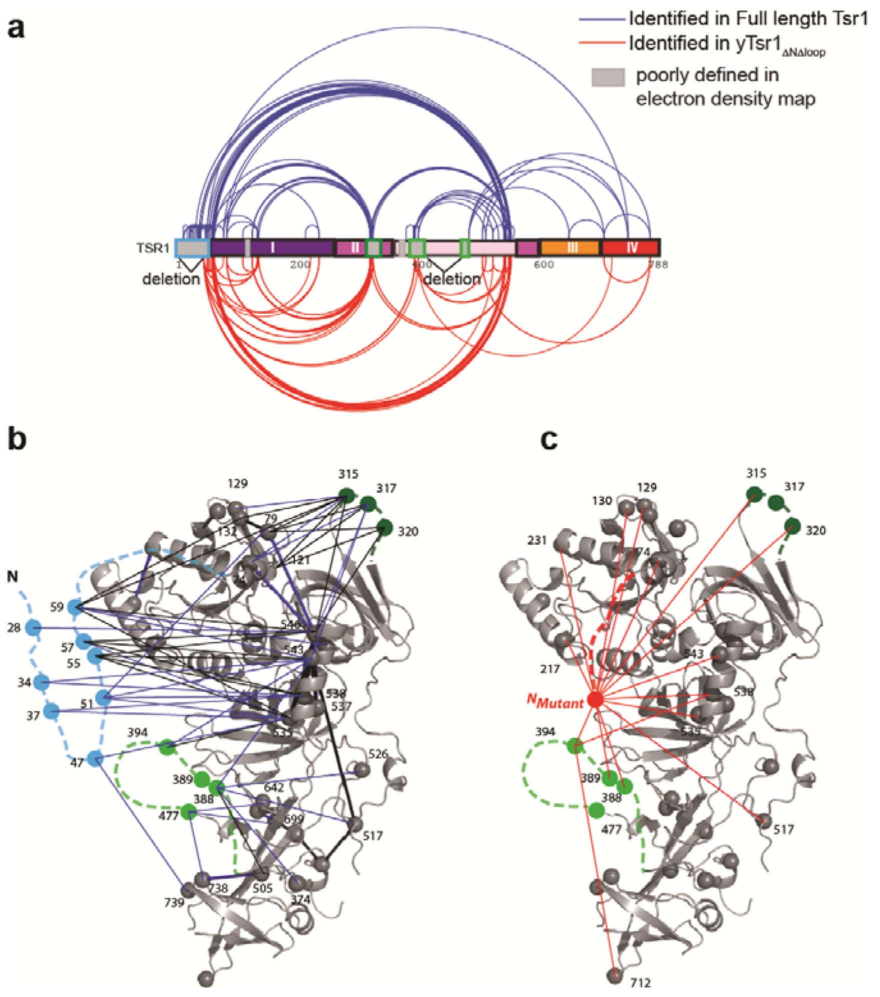

Supplementary Figure 3. Cross-link mass spectrometry of Tsr1

(**a**) Cross-linking/MS maps of full-length (blue arcs) and deletion mutant Tsr1 (red arcs). The linear sequence of Tsr1 is represented by boxes, colored according to domain. Regions that are not visible in the electron density map are gray boxes and sites of deletions are marked by triangles. (**b**) Back view of Tsr1 with Cα positions of residues that are cross-linked are shown as spheres. Loop regions that could not be modeled are shown with dotted lines with cross-linked residues represented by filled circles. Cross-links common to both Tsr1 species are black while those specific to the full-length protein are blue. (**c**) Cross-links found only in theTsr1$_{\Delta N\Delta loop}$ mutant dataset. The mutated N-terminus is marked in red.

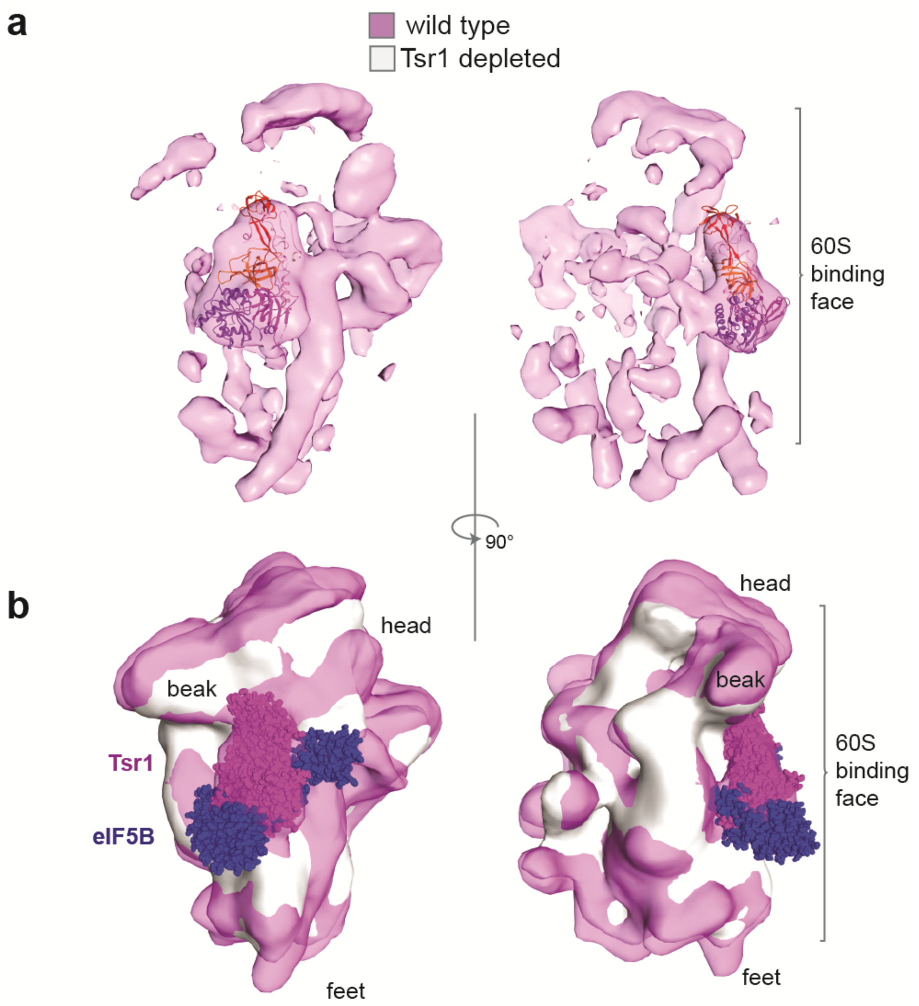

Supplementary Figure 4. Tsr1 and eIF5B occupy overlapping sites

(**a**) Difference map calculated by subtraction of EMD1926 from EMD1927 showing fit of Tsr1 molecule into the map. (**b**) Superposition of Tsr1 (magenta) and eIF5B (blue) bound to (pre-)40S particles; Tsr1 and eIF5B are depicted as sphere models.

Supplementary Methods

Cross-linking and Mass Spectrometry data acquisition

Respectively, 12 μg of purified Tsr1 and $Tsr1_{\Delta N\Delta loop}$ samples were cross-linked using BS3 with a 1:1 protein to cross-linker ratio at a final protein concentration of 0.3 M. The cross-linking reaction was incubated at 4 ºC for 2 hours and quenched with ammonium bicarbonate (final concentration 0.8 M) at 4 ºC for 30 mins. Cross-linked proteins were separated on 4-12 % NuPAGE Bis-Tris SDS-PAGE gels with MOPS running buffer (Life Technologies) and stained with Colloidal Blue stain (Life Technologies). Monomer bands of cross-linked Tsr1 and $Tsr1_{\Delta N\Delta loop}$ were excised, reduced, alkylated and then digested with trypsin[1]. A 6 μg aliquot of each sample was desalted on C18-StageTips[2] followed by LC-MS/MS analysis on a hybrid quadrupole-Orbitrap mass spectrometer (Q Exactive, Thermo Fisher). Peptides were loaded on a reversed-phase analytical column at 500 nl/min in 98 % mobile phase A (0.1 % formic acid in H_2O), 2 % mobile phase B (0.1 % formic acid, 80 % acetonitrile). Peptides were eluted at 200 nl/min over a linear gradient of 2 % to 40 % mobile phase B over 139 mins followed by a linear gradient to 95 % in 11 mins. Eluted peptides were sprayed directly into the Q Exactive spectrometer in data-dependent mode. For each acquisition cycle, the MS spectrum was recorded in the Orbitrap at 140,000 resolution. The ten most intense ions in the spectrum, with a precursor charge state ≥ 3+, were fragmented by Higher Energy Collision Induced dissociation (HCD). The fragmentation spectra were thus recorded in the Oribtrap at 35,000 resolution. Dynamic exclusion was enabled with single-repeat count and a 60 second exclusion duration.

MS Data analysis

Raw mass spectrometric data files were processed into peak lists using MaxQuant version 1.5.0.12[3] with default parameters, except that "Top MS/MS Peaks per 100 Da" was set to 20. Identification of cross-linked peptides was conducted using Xi software (ERI, Edinburgh). Peak lists of Tsr1 were searched against Tsr1 and reversed Tsr1 (decoy) sequences, while peak lists of $Tsr1_{\Delta N\Delta loop}$ were searched agains $Tsr1_{\Delta N\Delta loop}$ and reversed $Tsr1_{\Delta N\Delta loop}$ sequences. Search parameters were as follows: MS accuracy 6 ppm; MS2 accuracy, 20 ppm; enzyme, trypsin; specificity, fully tryptic; allowed number of missed cleavages, four; cross-linker, BS3; fixed modifications by BS3 that are hydrolyzed or amidated. Linkage specificity of BS3 was assumed to be for lysine, serine threonine, tyrosine and protein N-termini. We only accepted identified cross-linked residue pairs with an estimated 5 % false discovery rate (Fischer et al, submitted).

Supplementary References

1 Maiolica, A. *et al.* Structural analysis of multiprotein complexes by cross-linking, mass spectrometry, and database searching. *Mol Cell Proteomics* **6**, 2200-2211, doi:10.1074/mcp.M700274-MCP200 (2007).

2 Rappsilber, J., Ishihama, Y. & Mann, M. Stop and go extraction tips for matrix-assisted laser desorption/ionization, nanoelectrospray, and LC/MS sample pretreatment in proteomics. *Anal Chem* **75**, 663-670 (2003).

3 Cox, J. & Mann, M. MaxQuant enables high peptide identification rates, individualized p.p.b.-range mass accuracies and proteome-wide protein quantification. *Nat Biotechnol* **26**, 1367-1372, doi:10.1038/nbt.1511 (2008).
